# Cardiovascular Risk in Patients with Inflammatory Bowel Diseases—The Role of Endothelial Dysfunction

**DOI:** 10.3390/diagnostics14161722

**Published:** 2024-08-08

**Authors:** Maria A. Livzan, Galiya R. Bikbavova, Natalya S. Lisyutenko, Alisa E. Romanyuk, Oxana M. Drapkina

**Affiliations:** 1 Department of Faculty Therapy, Omsk State Medical University, 644099 Omsk, Russia; mlivzan@yandex.ru; 2 Department of Internal Medicine and Endocrinology, Omsk State Medical University, 644099 Omsk, Russia; n.labuzina@mail.ru; 3 Faculty of Medicine, Omsk State Medical University, 644099 Omsk, Russia; romalisa00@mail.ru; 4 National Medical Research Center for Therapy and Preventive Medicine, 101990 Moscow, Russia; drapkina@bk.ru

**Keywords:** inflammatory bowel disease, ulcerative colitis, Crohn’s disease, endothelial dysfunction, gut–vascular barrier

## Abstract

Inflammatory bowel disease (IBD) is associated with an increased risk of cardiovascular disease (CVD). Cardiovascular pathology in people with IBD has not been well studied to date, and a direct link between cardiovascular events and IBD has not been established. The mechanisms underlying this association include the parallel and dynamic interaction of inflammation, modulation of the composition of the gut microbiota, endothelial dysfunction, thrombogenicity, and increased endothelial and epithelial permeability. Endothelial dysfunction is a common aspect of the pathogenesis of IBD and atherosclerotic CVD and can be considered one of the most important factors leading to the development and progression of cardiovascular pathology in patients with IBD. The purpose of this literature review is to describe the mechanisms underlying the development of endothelial dysfunction and disorders of the structure and function of the gut–vascular barrier in the pathogenesis of the cardiovascular manifestation of IBD.

## 1. Introduction

Over the last century, there has been a shift in the profile of prevailing diseases, with infectious diseases gradually being replaced by non-communicable chronic diseases. An important characteristic of a modern patient is comorbidity, which A.R. Feinstein defined as “an additional clinical condition that existed or arose in the course of the patient’s ‘main’ disease” [1].

Multimorbidity is associated with higher rates of mortality, disability, side effects of treatment, increased use of health system resources, and lower quality of life [2]. Understanding how to treat people with multiple chronic diseases effectively is one of the most challenging problems modern healthcare faces [3]. The concept of associative multimorbidity has emerged in recent years. It is the non-accidental occurrence of a particular set of diseases. These diseases occur together more often than would be expected by chance [4].

Since the middle of the 20th century, there has been a steady increase in the incidence of inflammatory bowel disease (IBD) worldwide [5]. IBD includes ulcerative colitis (UC) and Crohn’s disease (CD) [6]. These are two heterogeneous, incurable, persistent, recurrent conditions characterised by non-infectious chronic immune-mediated inflammation of the colonic mucosa in patients with UC or transmural lesions of any part of the gastrointestinal tract in patients with CD [7]. The increase in the number of IBD patients has been attributed to factors such as the “westernisation” of lifestyles and diets [8]; the availability and widespread use of endoscopic diagnostic methods; and an increase in the birth rate since 1946 [9]. Despite the continued increase in the incidence of UC and CD, there is a decrease in the mortality of IBD patients associated with disease activity, which is due to the use of biological therapies, increased access to specialised care, and the “treat-to-target” STRIDE II approach in the context of individual patient needs [10].

As the life expectancy of patients with IBD increases, the number of people with multimorbidity also increases [11], including the combination of IBD with atherosclerotic cardiovascular diseases (CVDs) [12]. For effective prevention of CVDs, it is important to assess cardiovascular risk (CVR) factors since the concept of their timely detection is basic in the implementation of preventive measures [13]. The pathology of the cardiovascular system in patients with IBD has not been well studied to date, and a direct link between cardiovascular events and IBD has not been established. There is increasing evidence that conventional CVR factors have a lower prevalence in patients with IBD [14]. Non-conventional CVR factors and systemic chronic inflammation, such as hyperhomocysteinemia, leukocytosis, anaemia, corticosteroid therapy, thrombocytosis, high levels of C-reactive protein, and the erythrocyte sedimentation rate, play a major role in the development of atherosclerotic CVD in patients with UC and CD [15,16,17].

## 2. How Often Do Cardiovascular Diseases Occur in Patients with IBD?

Thanks to medical innovations, scientific and technological progress, increased access to education and information, gender equality, and public health achievements, life expectancy on the planet is increasing, and it is projected that the number of people aged 65 and over will double, exceeding 1.6 billion in 2050 [18].

The IBD population is undergoing similar demographic changes [19]. Elderly patients with IBD form the fastest-growing group [18] with more than one-third of the total number of patients [20,21].

Most studies, systematic reviews, and meta-analyses [22,23,24,25,26] support the view that IBD has a negative prognosis for the development of CVD, as reflected in the ECCO Guidelines on Extraintestinal Manifestations in IBD [27], which states that “there is a slightly increased risk of cerebral circulatory disorders, coronary heart diseases, mesenteric ischemia, atrial fibrillation and heart failure in patients with IBD compared to the general population, regardless of IBD therapy. No differences in cardiovascular disease mortality were observed”.

In a meta-analysis by H.H. Sun et al. [28], it was shown that patients with IBD have an increased risk of CVD, with the risk being higher in women, but CVD mortality was not increased in patients with either UC or CD. In the study by Alain Bitton et al. [29], it is stated that mortality from all causes is higher in populations with CD and UC than in the general population. Nevertheless, over the 10-year period studied by the scientists, a downward trend in the standardised mortality rate from various causes was revealed. In the study by Pia Manninen et al. [30], it was shown that patients with UC have a mortality rate similar to or slightly higher than that of the general population. But before concluding that IBD is not associated with CVD mortality, it is necessary to take into account that IBD comprises a heterogeneous group of people in terms of inflammatory activity and treatment. Studies stratifying patients by disease course, lesion extent, duration, activity, and age at the debut of the disease could identify subgroups of patients, with the highest risk of CVD and the associated risk of mortality [31]. In the study by A. Dregan [31], it was stated that patients with IBD had a higher death risk from CVDs.

## 3. The Endothelium Is the Structural Basis of the Circulatory System

The endothelium is made of flat cells of mesenchymal origin, lining the inner surface of blood and lymphatic vessels, as well as cardiac cavities. The mass of endothelium is estimated at 1.5–1.8 kg with a length of 7 km, and its area is comparable to the area of a soccer field. Such analogies make it clear that the endothelium is a unique tissue with representation in all organs [32]. The structure of the endothelium is specific for each organ and corresponds to organ function; thus, the endothelium has several types that determine the peculiarities of its physiological permeability. These include the solid type, fenestrated type, and sinusoidal type [33].

Vascular endothelial cells are lined with glycocalyx, which is a gel-like supramembrane complex consisting of many components including proteoglycans, glycoproteins, and proteins associated with the surface of the endothelium or located relatively freely in the supercellular space between the membrane of the endothelial cell and the liquid part of the blood. Proteoglycans are syndecans 1, 2, and 4, glipican, and perlecane; the major glycosaminoglycans are heparan sulphate, chondroitin sulfate, and hyaluronic acid; and glycoproteins include three families of adhesion molecules, the selectin family, the integrin family, and the immunoglobulin superfamily, and their expression depends on the surrounding microenvironment [34]. Glycocalyx is the primary link that is affected by hydrodynamic forces and provides mechanosensory functions. In addition, it regulates vascular permeability and the adhesion of immunocompetent cells and is a reservoir (binding and accumulation zone) for biologically active molecules, thus affecting the microenvironment and metabolic processes [35]. Changes in the thickness and composition of the endothelial glycocalyx relate to various pathological conditions, such as sepsis, hypertension, and diabetes mellitus [36,37]. Currently, the nature and degree of changes in endothelial glycocalyx in many diseases have not been fully studied, but this area of research is promising in terms of developing new therapeutic goals, including the treatment of atherosclerosis [38].

Endothelial cells differ from each other in orientation relative to the axis of the vessel, shape, size, and properties according to their hemodynamic and metabolic environment. [39]. A distinguishing morphological feature of endotheliocytes is the presence of specialised structures, including caveoles, vesicles, vesiculo-vacuolar organelles, transendothelial channels, and fenestrae. Large molecules such as albumin pass through the endothelium using caveoles and vesiculo-vacuolar organelles. It is considered that the permeability of capillaries is provided mainly by caveoles, which can transfer substances from the luminal to the basolateral surface of the cell. Vesiculo-vacuolar organelles, as their name suggests, are the result of the fusion of vesicles and vacuoles that form a channel through the endotheliocyte. Their diameter is usually larger than the diameter of caveoles. The most important element stabilising caveoles and vesiculo-vacuolar organelles is the protein caveolin. Currently, three proteins of the caveolin family have been identified including caveolin-1, -2, and -3 (Cav-1, -2, -3). It has also been established that Cav-1 and -3 are necessary for the formation of caveoles, while Cav-2 cannot independently provide caveogenesis but interacts with Cav-1 [40]. It is known that small molecules are transported through receptor-mediated transcytosis, or through fenesters if the endothelium is fenestrated. Fenestra are holes through the endothelial cell closed by a protein diaphragm. The key protein of this diaphragm is plasmalemmal vesicle protein-1 (PV-1). Thus, fenestra are not permeable structures.

Intercellular contacts are another mechanism of transporting substances through the endothelium. The endothelium has the same types of intercellular contacts as the epithelium, except for desmosomes. There are slot contacts, an adhesion zone, and tight contacts (TJ). The main protein of the adhesion zone in endotheliocytes is tissue-specific vascular endothelial cadherin (VE-cadherin), and in TJ, the most specific protein is claudin 5.

Another endothelium-specific formation is thought to be the presence of Weibel–Palade bodies, which are secretory vesicles containing von Willebrand factor (vWF) and several other proteins, P-selectin, tissue plasminogen activator, angiopoietin-2, and various cytokines [41].

**Figure 1:**
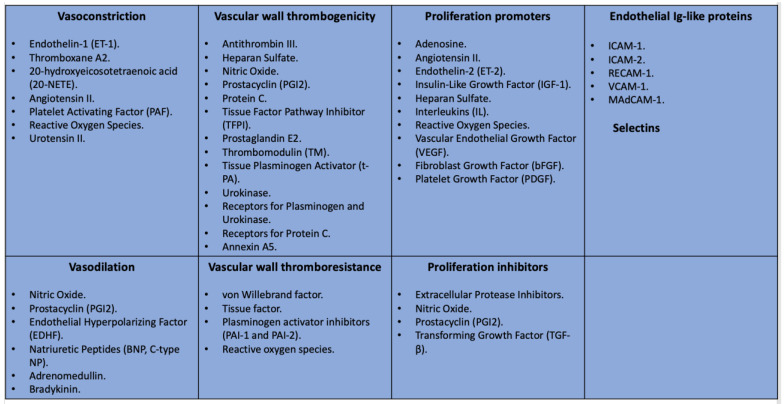
Main functions of the endothelium and mediators of endothelial dysfunction.

Recent studies have helped to clarify the role the endothelium plays in overall homeostasis [42]. The function of the endothelium is ensured by maintaining a balance between various biologically active molecules such as nitric oxide (NO), endothelin-1, vWF, the superfamily of cell adhesion molecules (CAMs), etc. (Figure 1).

Under physiologic conditions, the formation of anti-trombotic factors predominates over thrombogenic ones. The endothelium has a dilating effect on blood vessels, which prevents excessive permeability and the adhesion and aggregation of leukocytes and platelets, which is supported [43] by cytoprotective factors. These are high-density lipoproteins (HDL) [44], prostacyclin (PGI2), nitric oxide (NO), anticoagulants, antiplatelet agents, and fibrinolytics [42,45]. Bradykinin stimulates the release of NO and prostacyclin, enhancing antiplatelet effects [46]. However, several diseases, including chronic inflammatory conditions [47], metabolic disorders, smoking [48], and atherosclerosis [49], lead to endothelial cell (EC) dysfunction. Moreover, endothelial permeability, inflammation, and atherosclerosis have been shown to be inextricably linked.

Violation of EC homeostasis is the first stage of atherosclerosis since it leads to changes in vascular permeability, impaired blood clotting function, and lipid infiltration into perivascular intima, which causes damage to the endothelium [50]. The transport of lipoproteins through the barrier and into the subendothelial space plays a key role in the pathogenesis of atherosclerosis. Elevated circulating HDL levels are one of the best-characterised risk factors for ischemic heart disease (IHD), and LDL accumulation under the arterial endothelium is the first step in the pathogenesis of atherosclerosis [51,52]. Endothelial dysfunction (ED) occurs when there is an imbalance in the production or bioavailability of NO, obtained from the endothelium, which leads to a decrease in vasodilation and the initiation of prothrombotic and proinflammatory activity of the endothelium. During the inflammatory process caused by various factors, there is an increase in the production of interleukin (IL)-1, IL-6, TNF-α, and C-reactive protein (CRP), which generate an endothelial proinflammatory phenotype characterised by an increase in the expression of E-selectin, vascular cell adhesion molecules-1 (VCAM-1). and intercellular adhesion molecules-1 (ICAM-1). It has been established that caveolins are actively involved in the repair of the endothelial membrane and other functions in endothelial cells. Consequently, the loss of function of these proteins can contribute to the development of atherosclerosis. One of the studies on mice [53] showed conflicting data, namely, that genetic ablation of Cav-1 inhibits the progression of atherosclerosis by reducing LDL infiltration into the vessel wall, increasing NO production, and reducing the expression of leukocyte adhesion molecules, with a reduction in the area of atherosclerotic lesions of around 65%.

## 4. Gut–Vascular Barrier

Constant direct contact of the gastrointestinal mucosa with microbial and food antigens has led to the formation of mechanisms of protection against pathogenic microorganisms, commensals, and products of their metabolism. The intestinal barrier function is provided by a complex combining mucosal, epithelial, endothelial, and immune (innate and adaptive) barriers [54].

The intestinal mucosal barrier is represented by the following layers: the inner layer, which is virtually bacteria-free, and the upper layer, which contains microorganisms and products of their metabolism [55,56]. The composition of the gut microbiota depends on the dietary preferences of the host, genetic characteristics, and exogenous factors, such as antibiotics and the state of the immune system. It is associated with a wide range of diseases, the causal relationships of which are currently being discussed [57]. The wall mucus layer has antimicrobial properties and keeps bacteria away from the epithelium [58]. Enterocytes, the most represented type of colon epithelial cells, are interconnected by intercellular contacts (dense contacts—TJs, adhesive contacts—AJs, desmosomes) through which the throughput capacity is regulated [59]. Intercellular contacts are dynamic specialised structures consisting of transmembrane proteins such as 24 variants of claudins, occludin, tricellulin, three types of connective adhesion molecules (JAM-A, -B, -C), angulins, and intracellular proteins attached to the actin cytoskeleton, such as three types of zonula occludens (ZO-1,-2,-3) [60]. On the part of the microbiota, activity against epithelial cells is carried out through the production of a wide range of substances, ranging from enzymes synthesised by bacteria to short-chain fatty acids (SCFAs) [61]. SCFAs are especially important as they are the main source of nutrition for the intestinal epithelium and the most important regulatory factor in the production of antimicrobial proteins by the epithelium—beta-defensins [62,63]. The role of trimethylamine and its oxide, which is synthesised by some bacteria, in the development of atherosclerosis has recently been the subject of much debate [64]. Trimethcylaminoxide (TMAO) is formed in the human body from trimethylamine (TMA), a product of the processing of L-carnitine, choline, betaine, and lecithin by the intestinal microbiota. TMA penetrates through the intestinal wall by the mechanism of passive diffusion across enterocyte membranes and is transported to the liver via the portal circulation, where it is converted into TMAO. TMAO is currently regarded as a proatherogenic, proinflammatory, and prothrombotic metabolite [65]. In in vivo studies, it was found that the blood concentration of TMA and its oxide depends not only on the presence of microorganisms capable of producing TMA but also on the degree of permeability of the intestinal barrier [66]. El Hage R. et al. present data suggesting that high plasma TMAO levels are associated with insulin resistance, hyperlipidaemia, hyperglycaemia, and hyperexpression of inflammatory markers including TNF-α, IL-6, and C-reactive protein, which together lead to the development of atherosclerosis [67]. One study demonstrated a positive correlation between intima-media complex thickness and TMAO levels in individuals over 65 years. The same study indicated that the increase in TMAO levels in the blood was not only due to the consumption of large amounts of red meat, eggs, dairy products, and fish but was also mediated by a complex of factors [68]. Several research groups have shown that changes in the Firmicutes-to-Bacteroidetes ratio, and thus imbalances in microbial metabolites such as SCFA and TMAO, correlate with CVD pathogenesis [69,70,71].

The intestine is vascularised by arterioles and the capillary network, and microcirculation regulates oxygen and nutrient metabolism, homeostasis of tissue fluid, and recruitment of circulating leukocytes [72]. In the intestine, the endothelium is characterised by a fenestrated type, making it permeable to molecules up to 4 kDa in size. In recent years, intestinal endothelial cells have been emphasised as an additional element involved in regulating the intestinal barrier [73].

Epithelial cells, ECs, intestinal pericytes, and enteric glial cells participate in the formation of the gut–vascular barrier (GVB) [74]. Under physiological conditions, the EC layer lining the vessel lumen provides an anti-adhesive and selectively permeable barrier. ECs in the intestine, like enterocytes, are bound by protein AJs (support the function of the vascular barrier and control its permeability to molecules with high molecular weight) and TJs (control permeability to ions and small molecules <800 Da). AJs consist of the so-called vascular “guard of the endothelial barrier” cadherin (VE–cadherin). It is specific for ECs, α- and β-catenin, the formation of which precedes and is necessary for the formation of TJs. Disruption of AJs leads to the disassembly of TJs [75]. In intestinal ECs, TJs are formed by occludin, ZO-1, cingulin, the connective adhesion molecule-A (JAM-A), and claudins, the most specific for ECs is claudin-5 [76]. Increased permeability is associated with an increase in the flow of water, low molecular weight substances, as well as blood proteins—albumin, globulins, and fibrinogen—outside the vessel. The endothelium is also permeable to leukocytes, which pass either transendothelially or among endotheliocytes.

It has been shown that the most important stimuli that increase endothelial permeability are histamine, bradykinin, thrombin, vascular endothelial growth factor (VEGF), and angiopoietin-2 (Ang-2) [77]. Other factors for endothelial activation, with a longer duration of action in the case of intestinal inflammation [73], are lipopolysaccharides and increased production of proinflammatory cytokines IFN-γ, IL-1β, and TNF-α, which is accompanied by the expression of TLR, E-selectin, VCAM-1, and ICAM-1. It is assumed that under the influence of damaging factors, an increase in the permeability of the endothelium occurs earlier than that of the epithelium. Increased permeability of intercellular contacts of the epithelium leads to the entry of antigens from the external environment (intestinal lumen) into the subepithelial space and into the blood, which is accompanied by activation of the immune response and associated complications [56].

## 5. Endothelial Dysfunction and Changes in the Permeability of the Gut–Vascular Barrier—The Pathogenetic Link between IBD and CVD

Endothelial dysfunction (ED) is a common aspect of the pathogenesis of IBD and atherosclerotic CVD. This makes it one of the most important factors in the development and progression of cardiovascular pathology in patients with UC [78].

In patients with IBD, intestinal barrier dysfunction evolves under the influence of a complex of factors (genetic, microbial, dietary). An increase in its permeability promotes the translocation of microorganisms and products of microbial origin into the mucosal layer and intestinal epithelium, which leads to the activation of the immune response. Bacterial endotoxins that enter the bloodstream because of increased intestinal permeability have a damaging effect on the vascular endothelium, and as a result, its normal functioning is disrupted and ED occurs. According to modern studies, IBD is associated with the impaired formation of vasoactive substances, a change in the formation of thrombogenic and atrombogenic endothelial factors, hyper- or hypo-expression of endothelial adhesion molecules, increasing expression of proinflammatory cytokines, excessive formation of angiogenic factors, and a change in the sensitivity of ECs to angiogenic factors; thus, all mechanisms of endothelial damage are involved in the pathogenesis of UC and CD [79].

In a meta-analysis [80], IBD was shown to be associated with ED, as confirmed by instrumental studies involving 2330 patients with IBD and 2032 individuals of the control group. However, regression models demonstrated that disease activity, disease duration, and treatment do not significantly affect carotid intima-media thickness in patients with IBD. It should be noted that these patients had significantly lower serum levels of total cholesterol (TC), LDL, and HDL than those in the control group. Another meta-analysis [81] also showed that patients with IBD had significantly lower levels of TC, HDL, and LDL compared with healthy individuals of the control group; patients with CD had significantly lower levels of TC compared with UC patients; and the active IBD and non-mild UC groups had significantly lower levels of TC and LDL compared with the inactive IBD and mild UC groups, respectively. Such changes in TC, HDL, and LDL in patients with UC and CD may be explained by a reciprocal relationship between systemic inflammation of subgingival lipids and low intestinal absorption of cholesterol and fat in IBD patients [82].

Pro-inflammatory cytokines TNF-α, IL-1β, and IL-6 have been shown to be elevated in patients with IBD. The increased production of active oxygen species due to the increased expression of TNF-α and IL-6 leads to ED with an increased risk of CVD [83,84]. One study showed a significant increase in serum CRP levels in patients with IBD who were at increased risk of IHD [85]. A multicentre longitudinal study revealed that the effective control of inflammation, namely, the use of TNF-α inhibitors, reduces cardiovascular risk in patients with IBD, as evidenced by a reduction in aortic pulse wave velocity, which was used as a surrogate indicator of cardiovascular risk [86]. However, the use of TNF-α inhibitors is associated with an increase in triglyceride levels [87,88].

The link between IBD and venous thromboembolism (VTE) has been proven, and the mechanisms continue to be studied [89,90]. VTE is a specific feature of IBD, with an increase in IBD patients independent of traditional factors such as hospitalisation, surgery and postoperative period, bed rest, use of oral contraceptives, and hormone therapy [91]. High disease activity, especially over a prolonged period, is associated with the highest risk of VTE [92]. Researchers suggest that the role of microbial endotoxins in this regard is poorly understood. One study showed that the mesenteric vessel walls in patients with IBD had increased levels of plasminogen activator inhibitor of type 1 (PAI-1) and decreased levels of tissue-type plasminogen activator (t-PA) and urokinase-type plasminogen activator (u-PA) [93]. 

In addition, the role of hyperhomocysteinemia in the development of thrombosis in patients with UC and CD is equivocal. Hyperhomocysteinemia is an established risk factor for arterial and venous thrombosis and occurs in about 5% of the population [94]. The mechanisms underlying this observation are debated. One is associated with a decrease in NO synthesis by means of increased homocysteine levels and oxidative stress [95], which eventually leads to the development of ED and the formation of an atherosclerotic plaque [96]. According to a meta-analysis by Xiaoping Zhang et al., hyperhomocystenia is significantly more common in patients with IBD [97] than in individuals of control groups. The reason for this is a deficiency in vitamins B6, B12, and folic acid, which is often found in patients with IBD because of their poor diets, malabsorption syndrome, and medications [98].

NO is one of the most studied molecules in medicine. It has properties such as vasodilation, relaxation of spasmodic blood vessels, reduction of arterial pressure, and antiatherogenic effects [99]. Nitric oxide synthase (NOS), which catalyses NO synthesis, exists in different forms. Inducible NOS (iNOS) is expressed in response to inflammatory stimuli, and its expression is suppressed, for example, by glucocorticosteroids; endothelial NOS (eNOS) and neuronal NOS (nNOS) are constitutive NOS (cNOS). Under physiological conditions, cNOS generates low levels of NO, which have a direct regulatory effect. In contrast, iNOS generates high levels of NO, which mediates antimicrobial and antitumor activity [100].

Similarly, in the pathogenesis of IBD, these molecules are inflammatory mediators, vasodilators, antiplatelet agents, and protectors of the mucous membrane of the gastrointestinal tract [101]. Under chronic inflammation, proinflammatory cytokines are activators of iNOS synthesis. Its concentration increases significantly, and the effect becomes cytotoxic. It is believed that in conditions of acute inflammation, NO action has a protective effect, but chronic overproduction of NO has a pathological and damaging effect on the mucosa of the gastrointestinal tract, forming a vicious circle. Some studies have shown that NO production is reduced in patients with IBD [102,103], which leads to vasoconstriction due to decreased relaxation of smooth muscle cells and increased arterial stiffness, observed in patients suffering from chronic inflammation [104], potentiating increased production of vasoconstrictor endothelin-1 [105].

The detrimental role of excess NO in IBD patients has been debated as studies have shown higher levels of nitrite/nitrate in plasma, urine, and the lumen of the colon [106,107]. In addition, overexpression of iNOS has been shown to correlate with increased concentration of NO and disease severity [108].

Pathological angiogenesis is the culmination of maintaining chronic inflammation in IBD patients. The influx of angiogenic factors in inflammatory tissues is regulated by macrophages, mast cells, lymphocytes, and fibroblasts [109]. Hypoxia in the inflammatory zone also activates angiogenesis, increasing the release of vascular endothelial growth factor (VEGF) [110], fibroblast growth factor (bFGF) [111], and TNF-α [112,113]. 

With IBD, autoantigens initiate an immune response through adhesion molecules that mediate the interaction between endothelial and immune cells. Monocytes adhere to the vessel wall mainly by interacting with VCAM-1 and ICAM-1. Integrin α4β7 is a lymphocyte adhesion molecule that mediates the migration of the latter when interacting with the mucosal addressin cell adhesion molecule-1 (MAdCAM-1) [114]. MAdCAM-1 is expressed mainly in the intestine and promotes adhesion to the vascular endothelium of T and B cells, macrophages, and possibly eosinophils, basophils, and differentiated mast cells [115]. Patients with IBD are characterised by a significant increase in the level of adhesion molecules VCAM-1 and ICAM-1 in blood plasma [78]. Studies have shown that the expression of MAdCAM-1 is specific to intestinal vascular endothelial cells and significantly increases with CD [116]. Using data from another study, researchers [117] demonstrated that MAdCAM-1 is a sensitive marker of UC relapse and therapeutic efficacy. The use of humanised monoclonal IgG1 antibodies, which specifically bind to α4β7-integrin and selectively block its interaction with MAdCAM-1, is currently one of the most significant areas of targeted IBD therapy (vedolizumab) [115,118].

**Figure 2:**
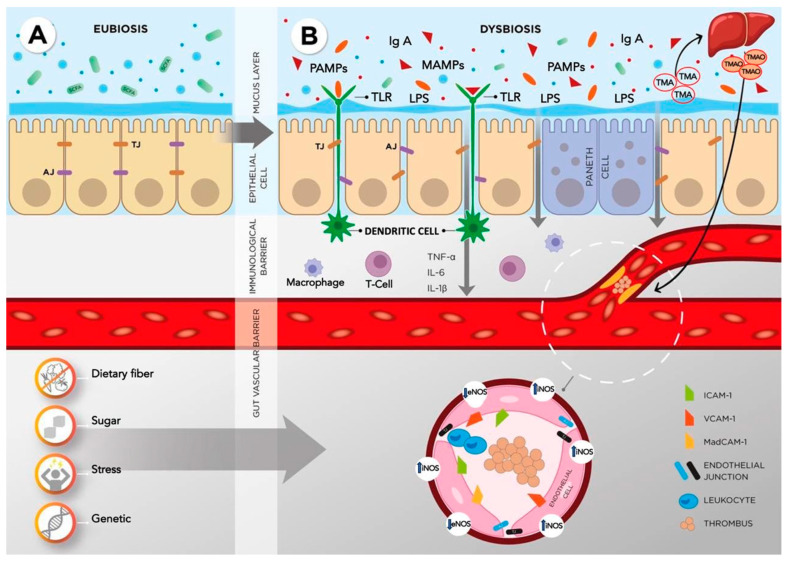
Endothelial dysfunction and changes in the permeability of the gut–vascular barrier—the pathogenetic link between IBD and CVD. List of abbreviations: AJ—adhesive contact, TJ—tight junction, PAMPs—pathogen-associated molecular patterns, MAMPs—microbe-associated molecular patterns, TLR—toll-like receptor, IgA—Immunoglobulin A, LPS—lipopolysaccharide, T-cell—T-lymphocyte, TNF-α—tumour necrosis factor-alpha, IL-6—interleukin-6, IL-1β—interleukin 1 beta, TMA—trimethylamine, TMAO—trimethylaminoxide, ICAM-1—intercellular adhesion molecule 1, VCAM-1—vascular cell adhesion molecule 1, MadCAM-1—mucosal vascular addressin cell adhesion molecule 1, iNOS—inducible NOS, eNOS—endothelial NOS. Left (**A**): In the normal intestine, barrier function is determined by the state of tight epithelial contacts and the quantity and quality of mucin protecting the epithelium. Right (**B**): under the influence of a combination of environmental, nutritional, and genetic factors, intestinal permeability and barrier function are compromised. The wall mucus layer has antimicrobial properties and keeps bacteria away from the epithelium. Enterocytes are interconnected by intercellular contacts (TJs, AJs, desmosomes) through which throughput capacity is regulated. M-cells capture microorganisms and present them to dendritic cells to recognise absorbed antigens, which ensures the formation of an immune response. Paneth cells secrete antimicrobial peptides. TMA is transported through the portal circulation to the liver, where it is converted into TMAO.

Figure 2 illustrates the described changes in the intestinal vascular endothelium and the gut–vascular barrier in patients with UC. All these mechanisms lead to increased permeability of the endothelial layer, the release of substances produced by the endothelium, imbalance of vasoconstrictors and vasodilators, adhesion and then diapedesis of leukocytes, increased smooth muscle tone, and procoagulant activity in patients with UC and CD [119].

Epithelial cells, ECs, pericytes, and enteric glial cells participate in the formation of the gut–vascular barrier. Under physiological conditions, the EC layer lining the vessel lumen provides an anti-adhesive and selectively permeable barrier. Intestinal ECs, like enterocytes, are bound by AJ and TJ proteins. Factors for endothelial activation are lipopolysaccharides and increased production of proinflammatory cytokines IFN-γ, IL-1β, and TNF-α accompanied by increased endothelial permeability, expression of TLRs, E-selectin, VCAM-1, and ICAM-1. Under the influence of damaging factors, an increase in the permeability of the endothelium occurs earlier than that of the epithelium. The increased permeability of the intercellular epithelial contacts leads to the entry of antigens from the intestinal lumen into the subepithelial space and into the blood. This is accompanied by activation of the immune response and associated complications. Under conditions of chronic inflammation, proinflammatory cytokines also activate i-NOS synthesis.

## 6. Future Research Perspectives

Despite the undoubted success achieved in the treatment and prevention of CVD, morbidity and mortality from CVD continue to lead in many countries of the world, so any experimental and clinical studies aimed at studying the pathogenesis and molecular basis of CVD, including in patients with IBD, are relevant and in demand. The control of inflammation is an important goal in reducing the risk of atherosclerosis in patients with UC and CD. In general, exposure to treatment for IBD has not been associated with an increased risk of serious adverse cardiovascular events. Moreover, some classes of drugs, especially 5-aminosalicylic acid (5-ASA) [24], TNF-α inhibitors [120], and azathioprine [121,122], reduce the risk of arterial thrombotic complications. In a Danish nationwide population-based study by Rungoe et al., the risk of IHD was significantly lower among IBD patients taking 5-ASAs than among those not taking them. In addition, patients who took 5-ASA for a long time had an even lower risk of developing IHD [123]. However, experts are concerned about the risk of cardiovascular adverse events during treatment with drugs from the groups of Janus kinase inhibitors and sphingosine-1-phosphate (S1P) receptor modulators, which are often used in the treatment of IBD [124,125]. Tofacitinib has been associated with venous thrombosis and thromboembolism, arterial hypertension, and increased low-density lipoproteins and triglyceride levels, while S1P receptor modulators can lead to adverse events such as bradycardia, atrioventricular blockade, and arterial hypertension. The experts point out that these cardiovascular events are limited to specific groups of patients with existing CVR. Therefore, the most important preventive measures include maintaining IBD remission [126], regular examination, and timely prescription of appropriate treatment.

Researchers will need to answer the question of whether statins should be prescribed to IBD patients with normal TC and LDL levels. They will also need to continue to search for effective targets of anti-inflammatory therapy and evaluate their therapeutic efficacy in atherosclerosis.

Another important area for future research is to further investigate the mechanisms by which the gut microbiota and its metabolites interact with epithelial and endothelial barrier permeability in patients with IBD and CVD and to carefully study drugs aimed at restoring gut microbial balance and diversity, as they may be the future for treating both atherosclerosis and IBD.

The epidemiology, clinical presentation, and outcomes of CVD in patients with IBD compared with the general population and other chronic inflammatory diseases can be assessed through the maintenance of patient registries and interdisciplinary collaboration. This knowledge is essential for the development of IBD-specific CVD prediction models, screening, and organisation of medical care.

## 7. Conclusions

The key mechanism of CVD development in patients with IBD is a systemic inflammatory reaction, a change in the composition of the gut microbiota, and increased production of endotoxins, which play a role both in the development of atherosclerosis and the maintenance of IBD activity.

In the debut of the inflammatory process, any imbalance between pro- and anti-inflammatory mechanisms leads to its prolongation. Chronic inflammation results in the loss of vascular wall integrity, ED, changes in NO and CAM expression leading to capillary and vein remodelling, endothelial cell proliferation, hypercoagulability, angiogenesis, and increased permeability of the epithelial and endothelial barrier.

The clear sequence of pathophysiological processes has not yet been established but appears to be a parallel, sequential, and dynamic interaction.

Long-term immunomodulatory supportive therapy likely reduces cardiovascular risks in patients with IBD by suppressing chronic systemic inflammation and influencing platelet aggregation, endothelial function, and lipid profiles. Further research is required to obtain more data to determine the accurate sequence and depth of these effects.
